# One More Death from Visceral Leishmaniasis Has Gone by Unnoticed. What Can Be Done?

**DOI:** 10.1371/journal.pntd.0002082

**Published:** 2013-06-27

**Authors:** Greg Matlashewski, Ravindra Pandey, Vidyanand Das, Pradeep Das

**Affiliations:** 1 Department of Microbiology and Immunology, McGill University, Montreal, Canada; 2 Rajendra Memorial Research Institute of Medical Sciences, Indian Council of Medical Research, Patna, Bihar, India; Emory University, United States of America

In January 2010, a 35-year-old mother from the village Baniyapur in Bihar, the poorest and second most populous state in India, had been weak and very ill for months with high fever, anemia, and wasting. She finally travelled by train to New Delhi to seek medical help in a major hospital. She was diagnosed with visceral leishmaniasis (VL), the most common febrile vector-born disease in Bihar, and given treatment with miltefosine and sent home. But intervention came too late—upon returning to Bihar, she died, leaving behind four children and a husband. Yet just one kilometre from her home, was a primary health care centre where she could have gone months earlier for life-saving treatment with miltefosine.

*Leishmania donovani*, the pathogen responsible for VL, is transmitted by the bite of an infected sand fly, and is the second most fatal parasitic disease after malaria [1], [2]. There are an estimated 200,000 to 400,000 new cases every year worldwide, over half of which occur in Bihar, India [3]. Humans are the only reservoir for transmission in Southeast Asia and Africa, and as a result, the greatest risk factor for VL is living in the same household with someone who has the disease [2]. In the months following her death in Baniyapur, three of her children and her husband also came down with VL. They were all diagnosed, treated, and cured at the local community primary care health centre.

This is an example where five individuals contracted VL in a single household where sand flies bred and transmitted the disease from family member to family member, resulting in one death and enormous suffering by those who survived. If there was some basic knowledge of this disease in the village, this situation could have been avoided. This is also the case for other neglected tropical diseases throughout the developing world. Too often, available life-saving drugs do not reach the people who need them—resulting in a continuous cycle of transmission and loss of life.

In the case of VL, the solution seems simple. Start a community awareness program so that people are better aware of VL and other febrile diseases. However, it is not so simple. Resources are limited, and the scale of the problem involves thousands of villages and a population of 90 million people living in Bihar, the most heavily endemic state in India. Nevertheless, there are solutions to be considered that can more generally be applied to countries with limited resources.

The first consideration is to provide better support and recognition for implementation research. Although basic research to identify new drug targets is laudable, in the case of VL, effective drugs exist, and the burden of this disease can be substantially reduced today if these drugs reach the people who need them [4].

More specific to VL, Bihar and other states in India have initiated a program of training Accredited Social Health Activists (ASHAs) to oversee maternal and childhood health. ASHAs are women who live in the villages, receive monthly training sessions at local community health care centres, and are generally familiar with the families in their villages. Recently, the Indian Council of Medical Research, with support from Grand Challenges Canada, has started a pilot program to train ASHAs about VL and other febrile communicable diseases. Posters describing the disease and what to do in case of infection are distributed to the ASHAs and have now been placed in over 500 endemic villages. This type of training can be performed cost-effectively using existing human resources. If the family described above had seen one of these posters in Baniyapur or had contacted a knowledgeable ASHA, the tragic outcome would have been avoided. For this program to succeed, however, it is essential that training be carried out twice a year and that ASHAs be paid for every VL case they identify—similar to what they are paid for delivering a pregnant woman to the primary health care centre to give birth.

There are other things to consider. India continues to use DDT spraying for vector control, which is largely unrealistic and unsustainable since it must be done in every house every six months to be effective. A better solution is to improve the quality of the houses. This can be done by installing newly available durable wall lining containing an insecticide approved by the World Health Organization, deltamethrin. This lining essentially covers the entire inside walls of the house with a durable bednet-like material that can provide protection in place for up to four years. Implementing this approach would reduce transmission not only of VL but also of other vector-borne diseases, including lymphatic filariasis, malaria, and viral encephalitis, which are also present in Bihar. Installing wall linings would be easier, faster, and more cost-effective than building new brick houses and would replace DDT spraying. The cost of this approach is a consideration, but there are organizations, non-governmental organizations, and even the World Bank who could provide support. Currently, there are pilot studies underway in Bihar, India, and Bangladesh initiated by the World Health Organization Special Programme for Research and Training in Tropical Diseases using the deltamethrin-treated durable wall lining in VL-endemic villages. Had this wall lining been in the households of Baniyapur two years ago, there would have likely been no VL cases among the five family members.

Finally, the state and national governments have important roles to play. Chiefly, they must ensure that the now-available drugs and diagnostics are present in the community primary health care centres and that the local practitioners know how to use them. If people go for treatment but there are no drugs, then they will seek the advice of local unqualified practitioners, and the disease will remain imbedded in the communities. Until it is possible to control VL through vaccination, these achievable measures can help villages like Baniyapur avoid the tragedy of VL and could provide a model for other neglected diseases.
